# Replacement of a Calcified Aortic Valve in a Porcine Aortic Root with the Perceval Sutureless Bioprosthesis

**DOI:** 10.1055/s-0042-1757795

**Published:** 2022-12-20

**Authors:** Giosuè Falcetta, Federico Del Re, Stefano Pratali, Uberto Bortolotti

**Affiliations:** 1 Section of Cardiac Surgery, Department of Cardiac Thoracic and Vascular Surgery, University Hospital, Pisa, Italy

**Keywords:** aortic valve replacement, bioprosthesis, sutureless bioprosthesis

## Abstract

We report a 79-year-old patient who had aortic valve replacement (AVR) using a porcine aortic root. Due to degeneration of the porcine aortic valve, he required reoperation during which a heavily calcified porcine root and aortic annulus prevented insertion of any traditional bioprosthesis. AVR was achieved using a sutureless bioprosthesis, combined with mitral valve replacement. The present case confirms the feasibility and advantages of using sutureless valve implantation in complex and high-risk redo procedures.

## Introduction

Sutureless bioprostheses represent a valid alternative to stented valves in isolated aortic valve replacement (AVR) in patients with calcific aortic stenosis. 1 Among these devices, the Perceval S aortic prosthesis (PSP; LivaNova, Saluggia, Italy) has shown excellent clinical and hemodynamic outcomes up to 5 years from AVR. 2 Since sutureless bioprostheses have also been recently reported to facilitate complex and high-risk redo procedures on the aortic valve, 3 to further confirm this observation we report the use of a PSP as a particular valve-in-valve procedure in a patient with a degenerated and grossly calcified porcine aortic root requiring simultaneous mitral valve replacement.

## Case Presentation

A 79-year-old man had undergone in 2006 a modified Bentall procedure using a 27-mm Prima Plus porcine aortic root (Edwards Lifesciences, Irvine, CA). Eleven years later, he presented with recent onset of exertional dyspnea. He denied any previous episode of fever. He was in atrial fibrillation. The chest X-ray was negative, and routine blood tests were unremarkable.

A two-dimensional (2D) echo demonstrated preserved left ventricular function, moderate aortic regurgitation, and severe mitral incompetence. Coronary angiography revealed diffuse coronary artery disease without significant stenoses of the major branches. Computed tomography demonstrated marked calcification of the aortic root, with multiple calcific spots in the aortic arch and coronary arteries.

At reoperation, the heart was approached through a repeat median sternotomy and completely isolated by dissection of pericardial adhesions. After cannulation of the distal aorta and both venae cavae, moderately hypothermic cardiopulmonary bypass (CPB) was instituted, and the aorta was cross clamped and opened approximately 5 mm above the previous distal suture line. The heart was arrested with antegrade cold blood cardioplegia into the coronary buttons repeated every 30 minutes.

Through a left atriotomy, the mitral valve was replaced by inserting a 27-mm stented porcine bioprosthesis, preserving the entire posterior mitral leaflet.

At inspection, the aortic valve showed only pinpoint calcifications, dehiscence of one commissure, and no evidence of infection. Based on the pathologic intraoperative findings also replacement of the porcine aortic valve was considered necessary.

**Fig. 1:**
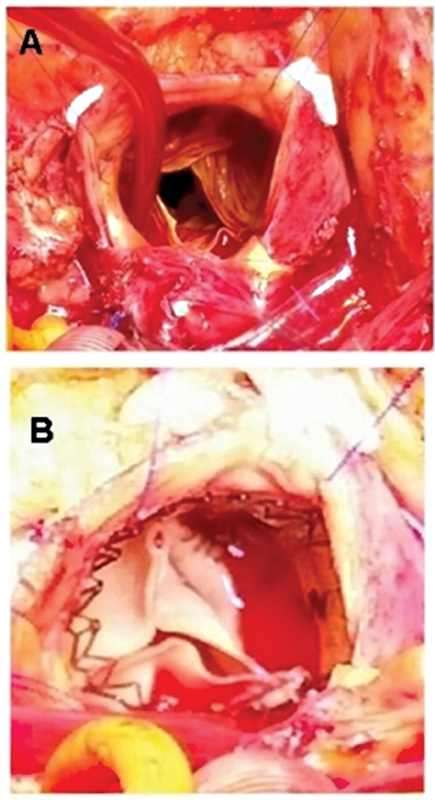
Intraoperative view showing ( **A** ) the incompetent aortic valve and ( **B** ) insertion of the Perceval sutureless bioprosthesis.

After excision of the cusps, the presence of a grossly calcified annulus prevented implantation of any standard bioprosthesis. Therefore, it was decided to implant an M size PSP ( Fig. 1 ). The total CPB time was 181 minutes, while aortic cross-clamp time was 157 minutes. The patient was weaned from CPB without difficulty, and the subsequent postoperative course was uneventful and without any complication.

Prior to discharge to a rehabilitation center, on postoperative day 8, a transthoracic 2D echo confirmed normal function of both prostheses, with a mean gradient of 12 mm Hg for the PSP. At 1-year follow-up, transthoracic 2D echo showed normal function of both mitral and aortic prostheses.

## Discussion

The use of sutureless bioprostheses for AVR, particularly the PSP, has been associated with gratifying overall results in the medium term. 1 One of the main demonstrated advantages of sutureless valve implantation has been the reduction of both cardiopulmonary and ischemic times, an advantage which is extremely valuable in high-risk patients and in those requiring combined procedures. 1 4 Feasibility of sutureless valve implantation in redo operations has been also demonstrated, and an International Expert Panel has recently included patients with prior AVR or other procedures 2 among the recommendations for sutureless valves. Indeed in many such patients, the use of a PSP as a valve-in-valve option may be a low risk and, at times, a life-saving strategy. 2

The patient herein reported presented with a degenerated Prima Plus porcine aortic root 11 years after prior AVR, also with concomitant mitral valve disease. Although symptoms were mostly related to the severe mitral regurgitation, the finding of mild cusp calcifications with dehiscence of one commissure in the porcine aortic valve suggested the need for concomitant AVR. The presence of a porcelain aortic root, with a severely calcified aortic annulus prevented AVR with any other stented or stentless bioprosthesis. The alternative of excising the entire root and performing a repeat Bentall operation was considered too challenging and at significantly higher risk, as previously highlighted. 4 In the present case, the availability of the PSP allowed us to find a comfortable solution to a difficult problem, also contributing to the reduction of the ischemic time in a complex reoperation in a patient requiring a combined procedure. Furthermore, the totally calcified porcine aortic valve ring provided an adequate landing zone for the PSP.

A recent review of the literature reported a total of 25 patients with a degenerated porcine aortic root or aortic homograft in whom the use of either a PSP or a rapid deployment bioprosthesis has proved effective in simplifying a challenging reoperation. 3 Moreover, the successful use of a PSP to overcome early failure of an aortic valve-sparing procedure has also been reported. 5

We believe that the present case confirms the efficacy of implanting a stentless bioprosthesis in complex and high-risk reoperations. This approach appears to be indicated in recipients of any type of biological conduit when tissue degeneration occurs, particularly widespread calcification of the aortic root.

## References

[1] Shrestha M Fischlein T Meuris B European multicentre experience with the sutureless Perceval valve: clinical and haemodynamic outcomes up to 5 years in over 700 patients Eur J Cardiothorac Surg 2016 49 01 234 241 25750010 10.1093/ejcts/ezv040

[2] Gersak B Fischlein T Folliguet T A Sutureless, rapid deployment valves and stented bioprosthesis in aortic valve replacement: recommendations of an International Expert Consensus Panel Eur J Cardiothorac Surg 2016 49 03 709 718 26516193 10.1093/ejcts/ezv369

[3] Vendramin I Lechiancole A Piani D Use of sutureless and rapid deployment prostheses in challenging reoperations J Cardiovasc Dev Dis 2021 8 07 74 34201997 10.3390/jcdd8070074 PMC8305208

[4] Minh T H Mazine A Bouhout I Expanding the indication for sutureless aortic valve replacement to patients with mitral disease J Thorac Cardiovasc Surg 2014 148 04 1354 1359 25260274 10.1016/j.jtcvs.2013.12.061

[5] Vendramin I Piani D Sponga S Bortolotti U Livi U Immediate failure of a valve-sparing procedure: repair with a Perceval sutureless prosthesis J Cardiovasc Med (Hagerstown) 2020 21 12 986 987 32639327 10.2459/JCM.0000000000001004

